# Analysis of AR/ARV7 Expression in Isolated Circulating Tumor Cells of Patients with Metastatic Castration-Resistant Prostate Cancer (SAKK 08/14 IMPROVE Trial)

**DOI:** 10.3390/cancers11081099

**Published:** 2019-08-01

**Authors:** Ivana Bratic Hench, Richard Cathomas, Luigi Costa, Natalie Fischer, Silke Gillessen, Jürgen Hench, Thomas Hermanns, Eloïse Kremer, Walter Mingrone, Ricardo Pereira Mestre, Heike Püschel, Christian Rothermundt, Christian Ruiz, Markus Tolnay, Philippe Von Burg, Lukas Bubendorf, Tatjana Vlajnic

**Affiliations:** 1 Institute of Pathology, University Hospital Basel, Schönbeinstrasse 40, 4031 Basel, Switzerland; 2 Department of Oncology/Hematology, Cantonal Hospital Graubünden, 7000 Chur, Switzerland; 3 Department of Oncology, Cantonal Hospital Winterthur, 8401 Winterthur, Switzerland; 4 Department of Oncology/Hematology, Cantonal Hospital St. Gallen, 9007 St. Gallen, Switzerland; 5 University of Bern, 3012 Bern, Switzerland; 6 Department of Urology, University Hospital Zurich, University of Zurich, 8091 Zurich, Switzerland; 7 Swiss Group for Clinical Cancer Research (SAKK) Coordinating Center, 3008 Bern, Switzerland; 8 Department of Medical Oncology, Cantonal Hospital Olten, 4600 Olten, Switzerland; 9 Clinic of Medical Oncology, Oncology Institute of Southern Switzerland, 6500 Bellinzona, Switzerland; 10 Department of Oncology/Hematology, Hospital of Solothurn, 4500 Solothurn, Switzerland

**Keywords:** mCRPC, circulating tumor cells, liquid biopsy, androgen receptor, ARV7

## Abstract

Despite several treatment options and an initial high response rate to androgen deprivation therapy, the majority of prostate cancers will eventually become castration-resistant in the metastatic stage (mCRPC). Androgen receptor splice variant 7 (ARV7) is one of the best-characterized androgen receptor (AR) variants whose expression in circulating tumor cells (CTCs) has been associated with enzalutamide resistance. ARV7 expression analysis before and during enzalutamide treatment could identify patients requiring alternative systemic therapies. However, a robust test for the assessment of the ARV7 status in patient samples is still missing. Here, we implemented an RT-qPCR-based assay for detection of AR full length (ARFL)/ARV7 expression in CTCs for clinical use. Additionally, as a proof-of-principle, we validated a cohort of 95 mCRPC patients initiating first line treatment with enzalutamide or enzalutamide/metformin within a clinical trial. A total of 95 mCRPC patients were analyzed at baseline of whom 27.3% (26/95) had ARFL+ARV7+, 23.1% (22/95) had ARFL+ARV7−, 23.1% (22/95) had ARFL−ARV7−, and 1.1% (1/95) had ARFL−ARV7+ CTCs. In 11.6% (11/95), no CTCs could be isolated. A total of 25/95 patients had another CTC analysis at progressive disease, of whom 48% (12/25) were ARV7+. Of those, 50% (6/12) were ARV7− and 50% (6/12) were ARV7+ at baseline. Our results show that mRNA analysis of isolated CTCs in mCRPC is feasible and allows for longitudinal endocrine agent response monitoring and hence could contribute to treatment optimization in mCRPC.

## 1. Introduction

While most newly diagnosed prostate cancer (PC) patients have potentially curable localized disease, still a significant proportion of patients progress or present initially with locally advanced or the metastatic stage [1]. The backbone treatment of metastatic PC (mPC) is androgen deprivation therapy (ADT). While at first most mPCs are hormone-sensitive, virtually all mPC patients progress and develop resistance to ADT within a median time of approximately 12–18 months [2,3,4]. Therapeutic options for metastatic castration-resistant prostate cancer (mCRPC) include novel androgen receptor (AR) targeting drugs (abiraterone, enzalutamide), taxane chemotherapy, immunotherapy (sipuleucel-T), and bone tropic radioisotopes (radium-223). Current recommendations for treatment strategies mainly depend on patient characteristics, extent of metastatic disease, prior treatments, and symptoms, but there is no randomized data for the optimal therapy sequence [5]. There is a consensus that novel AR targeting drugs should be used in treatment for asymptomatic men with mCRPC progressing on or after docetaxel (without prior abiraterone or enzalutamide) [6].

Multiple mechanisms of resistance contribute to progression to mCRPC, with reactivation of the AR signaling pathway, mediated by AR amplifications, AR mutations, and expression of splice variants, being the most prominent one [7]. Androgen receptor splice variant 7 (ARV7) is one of the most abundant constitutively expressed splice variants found in PC [8]. It is a constitutively active isoform of the AR that lacks the ligand-binding domain yet retains its transcriptional activity in a ligand-independent fashion. ARV7 mRNA expression in biopsies is significantly upregulated in hormone-naïve and mCRPC patients in relation to levels found in healthy tissue [9]. More recently, Antonarakis et al. have shown that ARV7 mRNA expression in circulating tumor cells (CTCs) may predict poor response to enzalutamide or abiraterone [10,11]. Results from subsequent studies have confirmed a correlation between positive ARV7 status in CTCs and impaired clinical progression-free survival under treatment with enzalutamide or abiraterone [12]. CTCs can be tested not only for ARV7 expression, but can also be used to identify AR hotspot mutations that could further enhance the diagnostic use of liquid biopsies to optimize treatment [13]. In conclusion, liquid biopsies are likely to become a key diagnostic test for tailoring existing treatment strategies. Despite the promising data in the literature, blood-based ARV7 testing has still not entered routine clinical practice due to ongoing controversies and technical challenges [14,15]. It has been recently emphasized that robust clinical validation of such assays is required before their routine use [15].

We designed a prospective, multicenter, randomized, open-label, phase II two-arm trial with the main objective to assess the efficacy of a combination first-line treatment with enzalutamide and metformin as opposed to enzalutamide alone in mCRPC patients progressing under ADT (SAKK 08/14, IMPROVE, NTC02640534). In this part of the trial, we address the potential use of molecular CTC analysis to determine the potential impact of ARV7 expression on patient outcome. We used the AdnaTest^®^ ProstateCancerPanel ARV7 (Qiagen) (Adna ARV7 Test), which was modified and further developed since the seminal paper of Antonarakis et al. [11]. As this test was not yet commercially available at the time of trial start, we aimed in this pilot study to examine its analytical and clinical validity by quantification of AR full length (ARFL) and ARV7 expression in isolated epithelial cell adhesion molecule-positive (EpCAM+) CTCs. Second, we aimed to assess the feasibility to detect prostate-specific markers (prostate-specific antigen (PSA), prostate-specific membrane antigen (PSMA), ARFL, and ARV7) in EpCAM+ CTCs in mCRPC patients subjected to either enzalutamide alone or to the combination enzalutamide/metformin.

## 2. Results

### 2.1. Sensitivity and Specificity of the Adna ARV7 Test

Analytical validity of the Adna ARV7 Test was assayed by spike-in experiments. The specificity of the test was investigated by the cell lines DU145 and PC3 and the sensitivity by LNCAP and VCAP. Gene expression of PSA, PSMA, ARFL, and ARV7 before spiking the cultured cells was individually tested on all cell lines by qRT-PCR (Figure S1). While VCAP and LNCAP featured a high expression of PSA, PSMA, ARFL, and ARV7, DU145 and PC3 were negative for PSA, ARFL, and ARV7 mRNA. We observed minute mRNA levels for PSMA in PC3, but not in DU145. These data are in line with published data [16]. We examined the Adna ARV7 Test’s specificity by spiking 50 × 10^3^ PC3 and 50 × 10^3^ DU145 cells in 6 mL of healthy donor blood. In both cases, we detected high levels of GAPDH mRNA, but no expression of PSA, ARFL, or ARV7. In PC3-spiked samples, we observed PSMA expression while DU145-spiked samples were negative. Additionally, we ran the test directly on isolated DU145 RNA that had been processed with the AdnaTest Prostate Cancer Detect kit and obtained the same results as with DU145 cells (Table 1). These data are in concordance with expression profile data of the respective cell lines (Figure S1) and suggest a high specificity of the Adna ARV7 Test.

The sensitivity was tested by spiking various numbers of cells from LNCaP, LNCaP95, and VCaP into healthy donor blood (Table 1). ARV7 expression was detected in samples with five LNCaP95 cells (100%, 5/5 replicates), five and ten LNCaP cells (2/3 and 2/4 replicates, respectively) or five and ten VCaP cells (3/3 and 2/3 replicates, respectively). ARFL expression was detected in samples with 10 LNCaP cells (100%, 4/4, replicates), five LNCaP cells (67%, 2/3 replicates), five LNCaP95 cells (100%, 5/5), and with 10 and five VCAP cells (3/3 replicates each) (Table 1). The varying sensitivity in the background of different cell lines likely originates from the differences in ARFL/ARV7 expression levels in these cells, since LNCaP95 and VCAP express ARFL and ARV7 at higher levels than LNCaP (Figure S1).

PSMA expression was detected in all tested blood samples that were spiked with VCaP, LNCaP, and LNCaP95 cells, respectively. PSA expression was found in samples with five LNCaP cells and in two of five blood samples with five LNCaP95 cells. We did not detect any PSA expression in the majority of VCaP-spiked samples (Table 1). This was unexpected as others and we had demonstrated (Figure S1) that the VCaP cell line expresses large quantities of PSA [17]. We analyzed PSA, PSMA, ARFL, and ARV7 expression in blood samples of 10 healthy donors. In eight healthy donors (three females, five males), we did not detect any PSA, PSMA, ARFL and ARV7 expression in blood samples. However, in the remaining two (female) blood samples, we only found expression of ARFL (Table 1).

### 2.2. Cross-Laboratory Variability

Cross-laboratory variability between our laboratory and Qiagen R&D Laboratory (Hilden, Germany) was objectified in two ways. First, we used five spiked-in samples with five LNCaP95 cells and five healthy donor samples (Table 2). Second, we tested 14 patient samples (Table 3). Samples were exchanged between the two laboratories and analyzed independently. Both laboratories were blinded for the results of the other. In both analyses, cDNA derived from captured EpCAM+ CTCs was exchanged between the two laboratories and all downstream analyses were performed independently.

Data concordance of spiked-in samples was 100% with regard to PSMA, ARFL, and ARV7 expression. GAPDH concordance was 90% and PSA concordance 70% (Table 2). Data concordance between 14 patient samples for PSMA, ARFL, and ARV7 was 100%, and for GAPDH 92.86%. Concordance for PSA was 78% (Table 3). Both concordance tests indicated lower concordance (<80%) regarding PSA expression. We tested PSA and PSMA expression in these samples also with the AdnaTest Prostate Cancer Detect assay (Adna Detect). PSA concordance between Adna ARV7 Test performed in Lab1 or Lab2 and Adna Detect was 57% and 50%, respectively (Supplementary Table S1). Hence, PSA was no longer tested in subsequent experiments. While the concordance between the two laboratories for detection of PSMA mRNA with the Adna ARV7 Test was 100%, concordance between Adna ARV7 Test and Adna Detect was only 57.1% (8/14) (Supplementary Table S1). In all six discordant PSMA cases, PSMA expression was not detected with Adna Detect. These results indicate higher sensitivity of the Adna ARV7 Test for PSMA mRNA detection in comparison to Adna Detect. 

### 2.3. Reproducibility of the CTC Profile Measured at Baseline by the Adna ARV7 Test

To test the reproducibility of the Adna ARV7 Test, two subsequent blood samples were drawn from five patients. GAPDH, PSMA, ARFL, and ARV7 expression were analyzed and the results were compared (Table 4). In four patients, from whom blood samples had been taken at baseline, the data concordance was 100% for PSMA, ARFL, and ARV7. Patient 5 (P5), from whom blood samples had been taken at progression, gave different results. While the first blood sample had EpCAM+ CTCs expressing PSMA, ARFL, and ARV7, the second blood sample had no detectable EpCAM+ CTCs.

### 2.4. Patient Cohort

**Figure 1:**
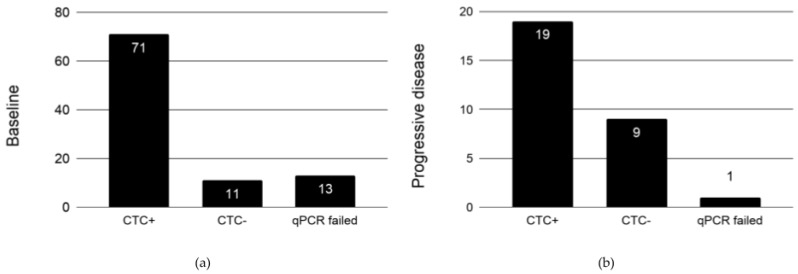
Analysis of circulating tumor cells (CTCs) at (**a**) baseline (*n* = 95) and (**b**) progressive disease (*n* = 29). Patient blood samples positive for CD45 and GAPDH expression, but negative for prostate cancer (PC)-specific markers (prostate-specific antigen (PSA), prostate-specific membrane antigen (PSMA), ARFL, and androgen receptor splice variant 7 (ARV7)) were scored as CTC negative. A patient blood sample is considered CTC positive if in addition to CD45 and GAPDH, at least one PC-specific marker is unambiguously detected. The number in each bar refers to the number of patients.

Based on our validation assay for the Adna ARV7 Test, we defined a scoring scheme for CTC analysis. Patient samples with CD45 and GAPDH expression, but negative for PC-specific markers (PSA, PSMA, ARFL, and ARV7) were scored as “no CTCs” or “CTCs negative for PC markers”. Samples with expression detection for either CD45 or GAPDH were excluded from further analysis. A total of 11 (11.6%) patients at baseline and 9 (31%) at progression had either “no CTCs” or had “CTCs negative for PC markers”. A total of 13 (13.7%) patient samples at baseline and one at the end of the treatment were excluded due to failed qPCR analysis (Figure 1).

### 2.5. Expression Profile of EpCAM+ CTCs in Patients at Baseline

**Figure 2:**
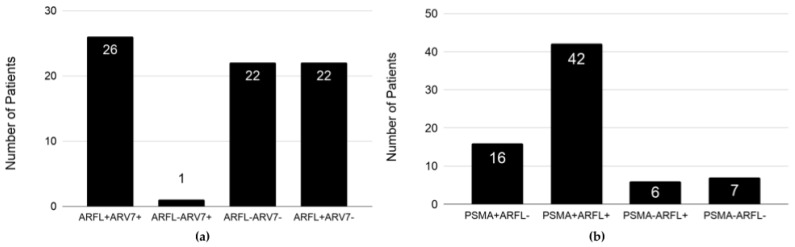
ARFL, ARV7, and PSMA expression in epithelial cell adhesion molecule-positive (EpCAM+) CTCs of metastatic castration-resistant prostate cancer (mCRPC) patients at baseline: (**a**) ARFL and ARV7 expression, and (**b**) PSMA and ARFL expression. The number in each bar refers to the number of patients with the indicated CTC phenotype.

At baseline, three major CTC phenotypes were observed at similar proportions: 26/95 (27.4%) of patients had ARFL+ARV7+, 22/95 (23.2%) had ARFL−ARV7−, and 22/95 (23.2%) had ARFL+ARV7− CTCs (Figure 2a). Only one of the patients had ARFL−ARV7+ CTCs. About half of the patients (42/95, 44.2%) had PSMA+ARFL+ CTCs, 16/95 (16.8%) had PSMA+ARFL−, 6/95 (6.3%) had PSMA−ARFL+, and 7.4% (7/95) had PSMA−ARF− CTCs at baseline (Figure 2b).

### 2.6. Expression Profile of EpCAM+ CTCs in Patients on Enzalutamide at Progressive Disease

**Figure 3:**
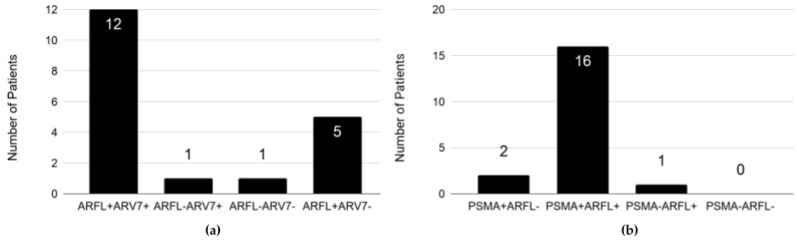
ARFL, ARV7 and PSMA expression in EpCAM+ CTCs of mCRPC patients at progressive disease (PD): (**a**) ARFL and ARV7 expression and (**b**) PSMA and ARFL expression. The number in each bar refers to the number of patients with the indicated CTC phenotype. The total number of analyzed patients with CTC-positive blood samples at PD was 19.

A total of 29/95 patients had another CTC analysis at progressive disease (PD), of whom 9 (31%) had no CTCs detected or their CTCs were negative for all tested mRNAs, 19 (65.5%) had CTCs positive for at least one PC-specific mRNA, and in one patient qPCR analysis failed. From 19 patients with evaluable CTCs data, the majority (12/19, 63.2%) had ARFL+ARV7+ and 5/19 (26.3%) had ARFL+ARV7− CTCs. ARFL−ARV7− and ARFL-ARV7+ CTC profiles were single observations, respectively (Figure 3a). 18/19 (94.7%) patients had positive CTCs for PSMA expression. From those 19 patients, 16/19 (84.2%) had PSMA+ARFL+, two patients (2/19, 10.5%) had PSMA+ ARFL−, and one patient had PSMA−ARFL+ CTCs (Figure 3b).

### 2.7. ARFL/ARV7 Conversions at Progressive Disease

To analyze conversions in ARFL and ARV7 expression, we only took patients (*n* = 25) into account who had evaluable data at both time points, baseline and at PD. 48% (12/25) were ARV7+ at PD. Of those, 50% (6/12) were ARV7+ and 50% (6/12) were ARV7− at baseline (Table 5). The majority of patients (13/17, 76.4%) with ARFL+ CTCs at baseline still had ARFL+ CTCs at progression. Interestingly, 5/25 patients with ARFL−ARV7− CTCs at baseline converted into all of the possible profiles: ARFL+ARV7+, ARFL−ARV7−, ARFL−ARV7+, ARFL−ARV7−, and “no CTCs”. Of three patients in whom we did not detect CTCs at baseline, two patients remained negative for CTCs, and in one patient we detected ARFL+ARV7− CTCs at PD (Table 5).

### 2.8. PSMA/ARFL Conversions at Progressive Disease

15 (out of 25) patients (60%) had PSMA+ARFL+ CTCs at PD. 13/17 (76.5%) patients who had PSMA+ CTCs at baseline retained PSMA expression at PD (Table 6). Three out of five patients who had PSMA- CTCs at baseline had PSMA+ CTCs at PD.

## 3. Discussion

The potential predictive value of ARV7+ CTCs in determining resistance to enzalutamide or abiraterone has been shown in several studies [10,11,18], although there remains some controversy since some patients with ARV7-positive liquid biopsies (CTCs or RNA from whole blood) might still derive benefit from enzalutamide or abiraterone therapy [19]. A recent prospective multicenter study strongly supports the clinical significance of ARV7 detection in CTCs as a prognostic marker, which is associated with shorter progression-free survival and overall survival (OS) [20]. Moreover, nuclear-specific localization of the ARV7 protein within the CTCs might be significant in terms of guiding treatment selection in mCRPC patients [20,21]. Despite these promising data pointing towards a predictive role of ARV7, a robust method to evaluate ARV7 expression in CTCs as part of routine clinical practice has not yet been established. Therefore, we assessed the performance of the Adna ARV7 Test in a series of spike-in experiments and in mCRPC patients from a multicenter, randomized, phase 2, open-label study investigating metformin effects on mCRPC patients during enzalutamide therapy (SAKK 08/14, IMPROVE). The Adna ARV7 Test enables CTC enrichment by EpCAM-based immunoisolation. Upon CTC enrichment, mRNA transcripts of prostate-specific markers (PSA, PSMA, ARFL, and ARV7) are quantified by RT-qPCR. There have been few studies using RT-qPCR for detection of ARV7 upon EpCAM-based CTC enrichment [10,11,22,23]. It has been demonstrated that the CTC detection rate of the custom modified AdnaTest and RT-qPCR is superior to the CellSearch assay, which is currently the only FDA-approved test for isolation of CTCs [24]. In patients with mCRPC, similar CTC detection rates between the custom modified AdnaTest, where CTCs were identified based on the presence of KLK3, PSMA or EGFR transcripts, and a digital droplet PCR assay detecting prostate-specific mRNA in whole blood, have been reported [24]. Despite slight differences between these approaches, all studies agree that RT-qPCR is sensitive enough to detect RNA transcripts in CTCs. In their seminal paper, Antonarakis et al. used a modified AdnaTest Prostate Cancer CTC Panel for CTC enrichment coupled with RT-qPCR analysis using custom-made primers for ARFL and ARV7. In addition, cDNAs for PSA, PSMA, and EGFR were analyzed by multiplex PCR and reaction products were quantified with an Agilent Bioanalyzer [11].

We tested the sensitivity and specificity of the Adna ARV7 Test by a series of spike-in experiments. We demonstrated high specificity for this test as we did not detect any expression of PSA, PSMA, ARFL, and ARV7 by using pure RNA isolated from DU145 cells, which are known to lack the expression of these markers. Moreover, no PSA, ARFL, or ARV7 expression was detected in samples spiked with approximately 50,000 PC3 cells and 50,000 DU145 cells. PSMA mRNA expression was observed in PC3 spiked samples (but not in DU145 spiked samples) as expected since we observed a low, yet detectable PSMA mRNA expression in the PC3 cell line (Figure S1). In healthy male donors, we did not observe any expression for any of the tested markers. However, in two of five healthy female donors, we detected ARFL expression solely in the blood samples. Expression of ARFL in bone marrow cells, platelets, and other tissues has been reported [25,26,27]. Of note, we detected CD45 expression in all healthy control and spike-in samples. This result is likely explained by leukocytes binding nonspecifically to the magnetic beads in the absence of CTCs.

Several studies have demonstrated an independent prognostic value of CTC counts for OS in mCRPC, where the presence of ≥5 CTCs per 7.5 mL of blood had an adverse effect [28,29,30,31,32]. Therefore, this threshold of ≥5 CTCs/7.5 mL of blood has been suggested to be used as a prognostic marker for OS [28,29,30,31,32]. For this reason, we performed a series of spike-in experiments with at least five cells from different cell lines in healthy female donor blood to test the sensitivity of our assay. We demonstrated that the sensitivity of the Adna ARV7 Test is at least five cells/6 mL of blood. However, we observed that the reproducibility of detecting five cells in at least 6 mL of blood varied between different cell lines, where LNCaP95 and VCaP cell lines outperformed LNCaP. This is most likely due to the lower expression levels of ARFL and ARV7 in LNCaP in comparison to VCAP, where the expression levels of ARFL and ARV7 are 10× and 40× higher, respectively (Figure S1). Additionally, in our manual spiking approach, we were not able to distinguish between dead and living cells that might explain the variability between replicates.

Next, we demonstrated a high interlaboratory concordance for GAPDH, PSMA, ARFL, and ARV7. In contrast, PSA results should be interpreted with caution due to high interlaboratory variability. Analysis of biological replicates showed good reproducibility (4/5 patients, 80%) of this assay, which has not previously been investigated by others.

Interestingly, we observed a dynamic change of ARV7 mRNA expression in the clinical study cohort during treatment with enzalutamide, as previously reported by Antonarakis et al. in mCRPC patients by a modified Adna Prostate Cancer Test [10]. At baseline, 28% of our mCRPC patients were ARV7+ before the start of first-line enzalutamide therapy, which is well in line with previous reports [10,11]. At PD, six out of eight patients retained ARV7 expression in their CTCs, while two reverted from ARV7+ CTCs at baseline to either ARV7− CTCs or “no CTCs” at progression. Conversion from ARV7+ CTCs to ARV7− CTCs was also observed by Antonarakis et al. in mCRPC patients by a modified Adna Prostate Cancer Test [10]. The reversions of ARV7 expression might occur due to an inactivation of the AR signaling axis, decreasing selective pressure for ARV7 expression. Alternatively, the mRNA levels might be below the detection threshold of the Adna ARV7 Test for biological or technical reasons. However, these assumptions remain to be proven in larger patient cohorts. Currently, the clinical trial is still ongoing and no final efficacy results are available. After completion of the trial, we will be able to correlate clinical data of all patients and determine the correlation between their CTC profiles and clinical outcomes.

PSMA is known to be over-expressed in advanced PC or mCRPC [33]. While strong PSMA expression in the tumor has been associated with higher tumor stages, Gleason Scores, preoperative PSA levels, HER2 expression, and a higher risk of biochemical recurrence [34], there is no correlation between blood PSMA mRNA and tumor stage, Gleason score, or serum PSA [35]. However, it has been shown that the detection of the PSMA mRNA in blood can predict biochemical recurrence after radical prostatectomy [35]. Furthermore, PSMA expression in CTCs is characterized by a high intra-patient heterogeneity and is found in a large portion (67%) of metastatic PC patients by the CellSearch^®^ assay [36]. Our data show PSMA expression in CTCs in the majority of the patients at baseline (58/95, 61%) and at PD (18/25, 72%). Moreover, PSMA expression in CTCs appears to be rather stable as the majority of the patients with PSMA+ CTCs at baseline retained PSMA+ CTCs (13/17, 76.4%) at progression.

The Adna ARV7 Test has certain restrictions that require consideration. First, there is no possibility to distinguish patients who do not have CTCs from patients where CTCs detection failed due to technical reasons. Second, it is not possible to evaluate whether PSMA, ARFL, and ARV7 mRNAs are expressed in the same cell. Finally, only EpCAM+ CTCs are analyzed, which might pose a selection bias as it is assumed that some CTCs decrease the expression of EpCAM as a result of epithelial–mesenchymal transition [37].

## 4. Material and Methods

### 4.1. Study Design

We aimed to prospectively evaluate the dynamics of PSA, PSMA, ARFL, and ARV7 markers on EpCAM+ CTCs from mCRPC patients during treatment with enzalutamide alone or in combination with metformin. Peripheral blood for CTC analysis was taken at baseline (before starting the therapy) and at the time of progression. The study was approved by the Swiss Group for Clinical Cancer Research (SAKK) Board (Bern, Switzerland) (2016-00127, 31 January 2016). All patients gave their signed informed consent before blood collection.

### 4.2. Patient Characteristics

A total of 95 asymptomatic or minimally symptomatic mCRPC patients with confirmed adenocarcinoma of the prostate progressing under ADT had been prospectively enrolled in the IMPROVE clinical trial (SAKK 08-14, Trials.gov: NCT02640534) between September 2016 and May 2019 and scheduled to undergo another line of therapy with either enzalutamide/metformin or enzalutamide alone. As patient recruitment is still ongoing, we are not allowed to present detailed information on the diagnostic characteristics of the enrolled patients at this point. Detailed patient characteristics at the time of registration and at progression are available in the Supplementary Material.

### 4.3. RNA Extraction from VCaP, LNCaP, PC3, and DU145 Cell Lines and Detection of PSA, PSMA, ARFL, and ARV7 Expression by RT-qPCR

Total RNA from VCaP, DU145, LNCaP, and PC3 cell lines was extracted with the RNeasy Mini Kit (Qiagen). RNA concentrations were determined using the NanoDrop ND-1000 spectrophotometer (NanoDrop Technologies). A total of 1.5 μg of total RNA was reverse transcribed into complementary DNA (cDNA) by using the SuperScript VILO cDNA Synthesis Kit (Invitrogen) according to the manufacturer’s instructions. Gene expression was examined by qPCR performed on 15× diluted cDNA samples using the QuantiNova SYBR Green Kit (Qiagen). All qPCR experiments were performed on QuantStudio 3 (Thermo Fisher). The threshold cycle (Ct) of PSA; PSMA, ARFL, and ARV7 expression was determined and normalized to that of human GAPDH to obtain a ΔCt value (Ct(gene of interest) − Ct(GAPDH)) from each sample. The relative mRNA expression level was calculated by normalizing the ΔCt to DU145 cells (2^−ΔΔCt^). GAPDH, PSA, PSMA, ARFL, ARV7 primers used for the qPCR analysis were obtained from the AdnaTest Prostate CancerPanel ARV7 (Qiagen) (Figure S1). Three biological replicates were analyzed for each cell line. The statistical significance in difference of expression levels was calculated with an ANOVA with a post-hoc Student’s *t*-test (two-tailed with assumptions of equal variances) followed by a Bonferroni correction.

### 4.4. Quantitative Real-Time PCR and PSA, PSMA, ARFL and ARV7 Detection in EpCAM+ CTCs

Patient’s peripheral blood samples for CTC analysis were collected in 8.5 mL ACD-A Tubes (BD Vacutainer, Becton Dickinson) before the start of the therapy and at the time of progression. All patient blood samples were stored at 4 °C immediately after withdrawal until further processing. CTC enrichment was performed within 30 h after blood draw by AdnaTest Prostate Cancer Select (Qiagen) according to the manufacturer’s instructions. AdnaTest Prostate Cancer Detect kit (Qiagen) was used to isolate mRNA from isolated CTCs and Sensiscript RT kit (Qiagen) for cDNA synthesis. cDNA was stored at −20 °C. Prior to qPCR, cDNA was pre-amplified by a Multiplex PCR Plus kit (Qiagen) as follows: Initial activation at 95 °C for 5 min followed by 18 cycles (95 °C for 30 s, 60 °C for 90 s, and 72 °C for 90 s). CD45, GAPDH, PSA, PSMA, ARFL, and ARV7 expression were determined by qPCR by the help of QuantiNova SYBR Green (Qiagen) on 20× diluted pre-amplified DNA samples. qPCR primers were provided by the AdnaTest Prostate CancerPanel ARV7 (Qiagen). All qPCR analyses were performed on QuantStudio 3 (Thermo Fisher). To rule out contamination, we analyzed patient samples along with their corresponding reverse transcriptase non-template controls. Data analysis was performed according to the manufacturer’s instructions. The assay that we used has two major differences from Antonarakis et al.’s method [11]. First, detection of RNA transcripts of PSA, PSMA, ARFL, and ARV7 in Adna ARV7 Test was performed exclusively by quantitative real-time PCR, while in Antonarakis et al.’s study PSA and PSMA transcripts were analyzed by an end-point PCR by Bioanalyzer. Second, while Antonarakis et al. determined absolute levels of ARFL and ARV7 by RT-qPCR, ARFL and ARV7 expression in Adna ARV7 Test was analyzed by RT-qPCR after an additional amplification by multiplex PCR.

### 4.5. Analysis of PSA and PSMA Expression in EpCAM+ CTCs by Adna Detect

The AdnaTest Prostate Cancer Detect kit (Adna Detect, Qiagen) was used to isolate mRNA from isolated CTCs and Sensiscript RT kit (Qiagen) for cDNA synthesis. cDNA was stored at −20 °C. cDNA was amplified by Multiplex PCR (Prostate Detect) for detection of PSA, PSMA, and actin as follows: Initial activation at 95 °C for 15 min followed by 42 cycles (94 °C for 30 s, 61 °C for 30 s, and 72 °C for 30 s) and a final step at 72 °C for 10 min. Actin served as an internal PCR control. Analysis of PCR products was performed with the Agilent 2100 Bioanalyzer (Agilent Technology) on a DNA 1000 Lab Chip according to manufacturer’s instructions. The sample was considered positive if the PCR band had a concentration ≥10 ng/μL and the control gene actin had been detected.

### 4.6. Cell Spiking—Validation Experiments

To determine the specificity and sensitivity of the Adna ARV7 Test for CTC detection, we performed a series of spiking experiments with defined numbers of cultured human prostate cancer cell lines that are known to either express PSA, PSMA, ARFL, and ARV7 (VCaP, LNCaP) or not (DU145, PC3). Cells were spiked into healthy donor blood. LNCaP, DU145, and PC3 cell lines were routinely passaged in Roswell Park Memorial Institute culture medium (RPMI 1640), and VCaP cell line in Dulbecco’s Modified Eagle’s Medium (DMEM) medium. All media were supplemented with 10% (DU145, PC3, VCaP) or 20% (LNCaP) fetal bovine serum (FBS) (Invitrogen), and 1% penicillin/streptomycin solution (BioConcept). Cell lines were cultured in a humidified incubator with 5% CO_2_ at 37 °C. For spiking experiments, all cell lines were cultured for at least two days after passaging and gently detached with detachin (Genlantis) and washed with phosphate buffered saline (PBS, Gibco). The spiked input cell numbers of 5, 10, and 68 cells were manually picked and transferred to 6 mL of peripheral blood of a presumably healthy female donor. The spiked input cell numbers of 200 and 50,000 were determined by dilution and transferred into 6 mL blood of a presumably healthy female donor. Blood samples with five LNCaP95 cells were kindly provided by Dr Siegfried Hauch, Qiagen, R&D Department, Hilden, Germany. Blood samples without spiked cells served as a negative control. Additionally, blood from a presumably healthy male donor (*n* = 5) was used to determine background levels of PSA, PSMA, ARFL, and ARV7 in peripheral whole blood. Spiked blood was stored under the same conditions as patient samples to minimize any bias and analyzed by a standard procedure that includes AdnaTest Prostate Cancer Select for CTC Isolation, AdnaTest Prostate Cancer Detect for mRNA isolation and cDNA synthesis, preamplification, and qPCR as described above (Section 4.4).

## 5. Conclusions

In summary, we comprehensively validated and helped to improve a commercially available EpCAM-based immunomagnetic enrichment method of CTCs in whole blood samples from mCRPC patients, aiming to assess mRNA expression of prostate-specific markers. To our knowledge, this is the first report on the Adna ARV7 Test in mCRPC patients. Apart from a few limitations, the Adna ARV7 Test provides an objective result of CTCs molecular profile without the need for CTC imaging and enumeration. The major advantages of this approach are the possibility to simultaneously assess the expression of several PC markers (PSMA, AR, and ARV7) on EpCAM+ CTCs and a straightforward workflow that is possible to establish in the majority of diagnostic laboratories.

## Figures and Tables

**Table 1:** The sensitivity and specificity of the Adna ARV7 Test.

Sample	GAPDH	PSA	PSMA	ARFL	ARV7
DU145 (50 × 10^3^)^3^	+	−	−	−	−
DU145 (RNA)^2^	+	−	−	−	−
PC3 (50 × 10^3^)^2^	+	−	+	−	−
Healthy ^10^ *	−	−	−	−/+	−
LNCaP (200)^1^	+	+	+	+	+
LNCaP (68)^1^	+	+	+	+	+
LNCaP (10)^4^	+	+	+	+	++−−
LNCaP (5)^3^	++−	+	++−	++−	++−
LNCaP95 (5)^5^	++++−	+++−−	+	+	+
VCaP (18 × 10^3^)^1^	+	−	+	+	+
VCaP (10)^3^	+−+	+−+	+	+	++−
VCaP (5)^3^	−	+−−	+	+	+

The number in superscript refers to the number of independent replicates. The number in brackets refers to the number of spiked cells. Detected gene expression level is marked with a plus, and absence with a minus. If the results between replicates differed, the result of each replicate is listed separately. * In has been detected.

**Table 2:** Adna ARV7 Test concordance between two independent laboratories. Sample set consisted of five healthy donor and five spiked-in samples containing five LNCaP 95 cells each. Numbers in brackets indicate replicates (number of samples with concordant results/total number of samples).

Laboratories	GAPDH	PSA	PSMA	ARFL	ARV7
Concordance	90% (9/10)	70% (7/10)	100% (10/10)	100% (10/10)	100% (10/10)

**Table 3:** Adna ARV7 Test concordance between two independent laboratories. Sample set consisted of 14 patient samples. Numbers in brackets indicate replicates (number of samples with concordant results/total number of samples).

Laboratories	GAPDH	PSA	PSMA	ARFL	ARV7
Concordance	92.86% (13/14)	78% (11/14)	100% (14/14)	100% (14/14)	100% (14/14)

**Table 4:** Adna ARV7 Test concordance between two independent blood samples from five patients. Numbers in brackets refer to the replicates (number of samples with concordant results/total number of samples).

Patients	PSMA	ARFL	ARV7
P1	100% (2/2)	100% (2/2)	100% (2/2)
P2	100% (2/2)	100% (2/2)	100% (2/2)
P3	100% (2/2)	100% (2/2)	100% (2/2)
P4	100% (2/2)	100% (2/2)	100% (2/2)
P5	50% (1/2)	50% (1/2)	50% (1/2)

**Table 5:** ARFL and ARV7 conversion at PD. Percentages refer to the fraction of patients within groups who at baseline shared biomarker constellation (first column). Numbers in brackets refer to the number of patients with specific ARFL/ARV7 profile analyzed at PD.

	Progressive Disease (PD)				
Baseline	ARFL+ ARV7+	ARFL+ ARV7−	ARFL− ARV7+	ARFL− ARV7−	no CTCs
ARFL+, ARV7+ (8)	75% (6)	12.5% (1)	0	0	12.5% (1)
ARFL+, ARV7− (9)	44.4% (4)	22.2% (2)	0	0	33.3% (3)
ARFL−, ARV7− (5)	20% (1)	20% (1)	20% (1)	20% (1)	20% (1)
no CTCs (3)	0	33.3% (1)	0	0	66.7% (2)
Total (25)	11	5	1	1	7

**Table 6:** ARFL and PSMA conversion at PD. Percentages refer to the fraction of patients within groups who at baseline shared biomarker constellation (first column). Numbers in brackets refer to the number of patients with specific PSMA/ARFL profile/total number of patients analyzed at PD.

	Progressive Disease (PD)				
Baseline	PSMA+ ARFL+	PSMA+ ARFL−	PSMA− ARFL+	PSMA− ARFL−	no CTCs
PSMA+, ARFL+ (14)	71.4% (10)	0	7.1% (1)	0	21.4% (3)
PSMA+, ARFL− (3)	33.3% (1)	66.7% (2)	0	0	0
PSMA−, ARFL+ (3)	66.7% (2)	0	0	0	33.3% (1)
PSMA−, ARFL− (2)	50% (1)	0	0	0	50% (1)
no CTCs (3)	33.3% (1)	0	0	0	66.7% (2)
Total (25)	15	2	1	0	7

## Supplementary Materials

The following are available online at https://www.mdpi.com/2072-6694/11/8/1099/s1, Figure S1: qPCR analysis of expression profiles of PSA, PSMA, ARFL and ARV7 genes in DU145, PC3, LNCaP, and VCaP cell lines; Table S1. PSA and PSMA expression detection concordance between two independent tests: Adna ARV7 Test and AdnaTest Prostate Cancer Detect.
