# Supplementary material for: Analysis of AR/ARV7 Expression in Isolated Circulating Tumor Cells of Patients with Metastatic Castration-Resistant Prostate Cancer (SAKK 08/14 IMPROVE Trial)
Source: Cancers (Basel). 2019 Aug 1;11(8):1099. doi: 10.3390/cancers11081099 (PMC6721786; doi:10.3390/cancers11081099)
Supplement: Supplementary file 1 [file cancers-11-01099-s001.pdf]

Supplementary Materials: Analysis of AR/ARV7 Expression in Isolated Circulating Tumor Cells of Patients with Metastatic Castration-Resistant Prostate Cancer (SAKK 08/14 IMPROVE Trial)

Ivana Bratic Hench, Richard Cathomas, Luigi Costa, Natalie Fischer, Silke Gillessen, Jürgen Hench, Thomas Hermanns, Eloïse Kremer, Walter Mingrone, Ricardo Pereira Mestre, Heike Püschel, Christian Rothermundt, Christian Ruiz, Markus Tolnay, Philippe Von Burg, Lukas Bubendorf and Tatjana Vlajnic; Swiss Group for Clinical Cancer Research (SAKK)

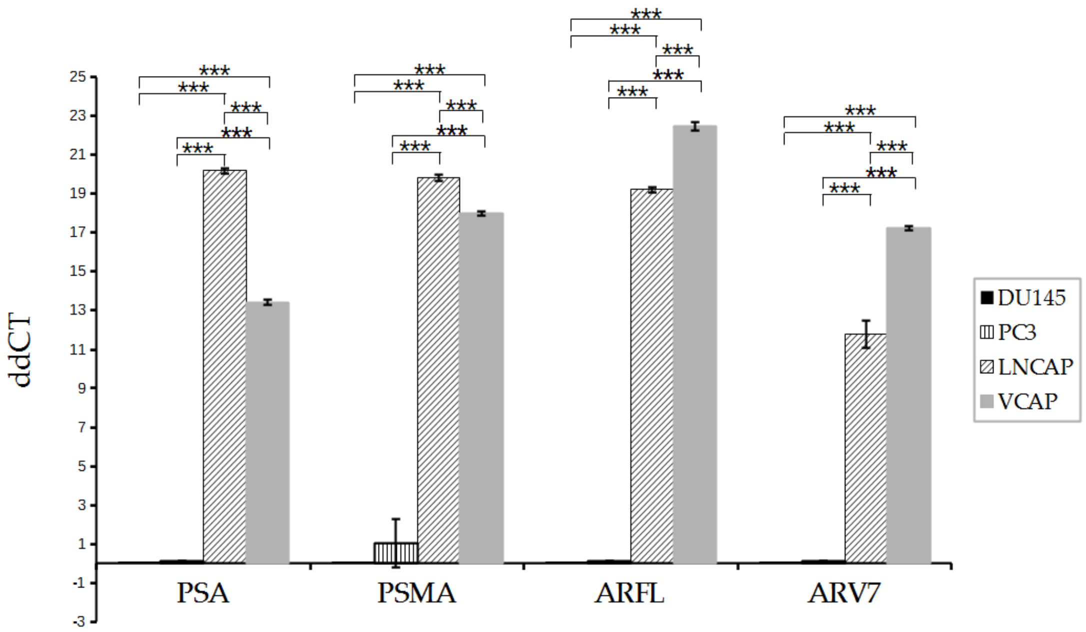

Figure S1. qPCR analysis of expression profiles of PSA, PSMA, ARFL and ARV7 genes in DU145, PC3, LNCaP, and VCaP cell lines. PSA, ARFL, and ARV7 mRNA expression was not detected in DU145 and PC3 cell lines. GAPDH normalized ddCT values over DU145 as means of three biological replicates are shown. Error bars represent standard deviations and asterisks statistical significance (ANOVA with post-hoc Student t-test (two-tailed with assumptions of equal variances) followed by Bonferroni corrections; *: $p \leq 0.05$, **: $p \leq 0.01$, ***: $p \leq 0.001$).

Table S1. PSA and PSMA expression detection concordance between two independent tests: Adna ARV7 Test and AdnaTest Prostate Cancer Detect. Sample set consisted of 14 patient samples. Numbers in brackets indicate the number of samples with concordant results/total number of samples. Cross concordance refers to the concordance between Adna ARV7 Test results of two independent laboratories and AdnaTest Prostate Cancer Detect.

Gene expression	Adna ARV7 (Lab1): Adna Detect	Adna ARV7 (Lab2): Adna Detect	Cross concordance
PSA	57.1% (8/14)	50% (7/10)	21.4% (3/14)
PSMA	57.1% (8/14)	57.1% (8/14)	57.1% (8/14)

Supplementary Methods:

Patient characteristics

95 asymptomatic or minimally symptomatic mCRPC patients with confirmed adenocarcinoma of the prostate progressing under ADT had been prospectively enrolled in the IMPROVE clinical trial (SAKK 08-14, Trials.gov: NCT02640534) between September 2016 and May 2019 and scheduled to undergo another line of therapy with either enzalutamide/metformin or enzalutamide alone. Before the next line of therapy, all patients had received ADT either with GnRH analogs or were subjected to the bilateral orchiectomy (medical or surgical castration). At the time of registration, all patients had PD defined by serum PSA level (minimum of two rising PSA levels and an absolute increase in total ≥ 2ng/ml with an interval ≥1 week between each determination), soft tumor (defined by RECIST 1.1) or bone disease progression (defined by Prostate Cancer Working Group 2 (PCWG2) guidelines with two or more new lesions on the bone scan) [1]. Patients were excluded if they received treatment with novel endocrine agents (abiraterone acetate, TAK-700, TAK-683, TAK-448, VT464, ODM201, ARN509), radioisotopes, tyrosine kinase inhibitors, and other small molecules, immunotherapy, or chemotherapy (with the exception of docetaxel chemotherapy in hormone-sensitive prostate cancer) prior to this trial.

Disease progression in the SAKK08-14 IMPROVE trial is defined by one of the following criteria: the presence of radiographic progression and symptomatic/clinical progression; the presence of radiographic progression and PSA progression, or the presence of symptomatic/clinical progression and PSA progression. Radiographic progression is defined by the presence of one of the following parameters: disease progression on bone scans or in soft tissue according to PCWG2 criteria. Symptomatic/clinical progression is defined by one of the following: occurrence of skeleton-related events (pathological fracture, spinal cord compression) due to bone metastases; the treating physician decides for intervention due to new disease-related complications (e.g., urinary obstruction, hydronephrosis); the treating physician decides to initiate new systemic anti-cancer therapy; or progressive disease-related pain despite consistent analgesic treatment. PSA progression is defined as ≥ 25% increase above baseline (at registration) and an increase in the absolute PSA value of ≥ 5 ng/mL in case if PSA levels had not decreased under treatment with the trial drug: ≥ 25% increase over the nadir and an increase in the absolute PSA value of ≥ 5 ng/mL in case when PSA response was < 50% under treatment with the trial drug; or ≥ 50% increase over the nadir and an increase in the absolute PSA value of ≥ 5 ng/mL in case when PSA response was ≥ 50% under treatment with the trial drug.

Reference

1. Scher, H.I.; Halabi, S.; Tannock, I.; Morris, M.; Sternberg, C.N.; Carducci, M.A.; Eisenberger, M.A.; Higano, C.; Bubley, G.J.; Dreicer, R.; et al. Design and end points of clinical trials for patients with progressive prostate cancer and castrate levels of testosterone: recommendations of the Prostate Cancer Clinical Trials Working Group. *J. Clin. Oncol. Off. J. Am. Soc. Clin. Oncol.* **2008**, *26*, 1148–1159.
